# Impact of exercise on oocyte quality in the POLG mitochondrial DNA mutator mouse

**DOI:** 10.1530/REP-18-0061

**Published:** 2018-06-05

**Authors:** Christine Faraci, Sofia Annis, Joyce Jin, Housaiyin Li, Konstantin Khrapko, Dori C Woods

**Affiliations:** Department of Biology Northeastern University, Boston, Massachusetts, USA

## Abstract

The mtDNA ‘mutator’ mouse, also called the ‘POLG’ mouse, is a well-characterized model frequently used for studies of progeroid aging. Harboring a mutation in the proofreading domain of the mitochondrial polymerase, polymerase-γ (*Polg*), POLG mice acquire mtDNA mutations at an accelerated rate. This results in premature mitochondrial dysfunction and a systemic aging phenotype. Previous work has demonstrated that the progeroid phenotype in POLG is attenuated following endurance exercise, the only reported intervention to extend health span and lifespan of these mice. Herein, oocyte quality was evaluated in sedentary and exercised POLG mice. In mice homozygous for the *Polg* mutation, litter size is dramatically reduced as compared to heterozygous *Polg* mice. Following ovarian hyper-stimulation, oocytes were retrieved until 9 months of age in exercised and sedentary groups, with no oocytes ovulated thereafter. Although ovulated oocyte numbers were not impacted by exercise, we did find a modest improvement in both the ovarian follicle reserve and in oocyte quality based on meiotic spindle assembly, chromosomal segregation and mitochondrial distribution at 7 months of age in exercised POLG mice as compared to sedentary counterparts. Of note, analysis of mtDNA mutational load revealed no differences between exercised and sedentary groups. Collectively, these data indicate that exercise differentially influences somatic tissues of the POLG mouse as compared to oocytes, highlighting important mechanistic differences between mitochondrial regulatory mechanisms in the soma and the germline.

## Introduction

In most female mammals, a natural consequence of aging is a reduction in the quantity and quality of oocytes, with the likelihood of successful pregnancy therefore substantially diminished over time. In humans, the aging-related decline in oocyte quality is linked to a dramatic risk of aneuploidy (Hassold & Chiu 1985, Battaglia* et al.* 1996, Broekmans* et al.* 2009, Tilly & Sinclair 2013). An accruing body of evidence from both animal models and clinical IVF data attribute aberrant mitochondrial function to many of the properties linked with the age-associated decline in oocyte quality (Dumollard* et al.* 2007, Broekmans* et al.* 2009, Bentov* et al.* 2011, Tilly & Sinclair 2013). In normal oocytes, mitochondria perform a diverse range of cellular functions that are essential for oocyte maturation and meiotic spindle assembly, fertilization and subsequent preimplantation embryogenesis (Van Blerkom* et al.* 1995, Igarashi* et al.* 1997, 2005, Schon* et al.* 2000, Eichenlaub-Ritter* et al.* 2004, Zheng* et al.* 2007, Bentov* et al.* 2010). In the early stages of the oocyte maturation process, prior to germinal vesicle break down, mitochondrial biogenesis ensures that the critical threshold number of mitochondria required for successful embryogenesis is met (Piko & Matsumoto 1976, Jansen & Burton 2004, Wai* et al.* 2010). Upon initiation of maturation to metaphase II (MII), mitochondrial biogenesis ceases and does not resume again in the developing embryo until post implantation (Piko & Taylor 1987, Reynier* et al.* 2001, El Shourbagy* et al.* 2006, Santos* et al.* 2006, Spikings* et al.* 2007, Wai* et al.* 2010). At this time, a subpopulation of mitochondria hyperpolarizes, resulting in an increase in mitochondrial membrane potential (Δψ_m_) and translocates to the perinuclear region to provide the ATP required for successful meiotic spindle formation and proper chromosome segregation (Van Blerkom* et al.* 2002). Additionally, a subset of mitochondria adjacent to the plasma membrane increases Δψ_m_ to mediate sperm penetration at the time of fertilization. In aged oocytes mitochondrial dysfunction becomes common, indicated by both reduction in Δψ_m_ and failure to localize to the proper perinuclear position, with mitochondrial aggregation frequently observed as a marker of poor oocyte quality (Wilding* et al.* 2001, Selesniemi* et al.* 2011). In addition to aberrant localization and Δψ_m_, the average number of mitochondria decreases on a per-oocyte basis, concomitant with decreases in ATP biosynthesis and tricarboxylic acid cycle metabolites and increases in irregular ultrastructural morphology and mtDNA mutational loads (Reynier* et al.* 2001, May-Panloup* et al.* 2005, Santos* et al.* 2006, Duran* et al.* 2011, Murakoshi* et al.* 2013, Simsek-Duran* et al.* 2013). Experimental evidence also points to mitochondrial dysfunction as a direct cause of poor oocyte quality. For example, in a mouse model for maternal diabetes, Wang *et al*. determined that poor reproductive outcomes mirrored those observed in aged oocytes, including abnormal mitochondrial ultrastructure, reduced ATP generation, meiotic spindle defects and improper chromosome segregation (Wang* et al.* 2009). Collectively, strong evidence points toward mitochondrial dysfunction as a root cause of oocyte failure with age.

The mtDNA ‘mutator’ mouse model, which harbors a D257A mutation in the exonuclease domain of the ‘proofreading’ DNA polymerase-γ (*Polg*) gene, exhibits a systemic multisystem premature aging phenotype attributed to accrual of mtDNA mutations and mitochondrial dysfunction (Trifunovic* et al.* 2004, Kujoth* et al.* 2005). Among the hallmark characteristics associated with aging, POLG-mutant mice acquire severe and accelerated onset of sarcopenia, hearing loss, osteoporosis, graying of fur and alopecia, thymic involution, testicular atrophy, enlarged heart, loss of red blood cells, weight loss, as well as a marked reduction in lifespan (Trifunovic* et al.* 2004, Kujoth* et al.* 2005). Importantly, a series of reports has now demonstrated that endurance exercise prevents the systemic aging phenotype in the POLG mouse (Safdar* et al.* 2011*a*,*b*, 2016*b*, Clark-Matott* et al.* 2015). POLG mice performing endurance exercise show remarkable phenotypic improvements in every tissue examined, and are in large visually indistinguishable from age-matched, WT counterparts (Safdar* et al.* 2011*a*). For example, exercise ameliorates the premature aging-related outcomes of the POLG mutator phenotype on sarcopenia, cardiomyopathy, brain atrophy, fat deposition and hemoglobin production. Additionally, molecular markers for mitochondrial biogenesis and function are upregulated in POLG mice following exercise, with levels comparable to WT mice (Safdar* et al.* 2016*b*). Taken alongside visually normal mitochondrial appearance and an absence of increase in oxidative damage in exercised POLG mice, the collective data indicate a marked improvement in mitochondrial quality in POLG mice on an exercise regime. Furthermore, in exercised POLG mice, lifespan is also extended, comparable to that of WT mice. Of note, it was also demonstrated that endurance exercise results in a significant increase in testicular mass and a modest increase in ovarian mass (Safdar* et al.* 2011*a*), although gonadal function was not examined. Herein, we describe the effect of the POLG mutator mouse phenotype at the level of the oocyte, using established markers of oocyte quality. Moreover, given the noteworthy impact of endurance exercise on the multisystem aging phenotype in the POLG mouse, we sought to determine whether an identical exercise regime confers similar benefits to oocytes in female POLG mice.

## Materials and methods

### Animals

Heterozygous mice (*Polg* ^D257A/+^) were obtained from the Jackson Laboratory and used to generate the homozygous knock-in mtDNA mutator mice (*Polg* ^D257A/D257A^). WT C57BL/6 mice were from Charles River. All experiments described herein were reviewed and approved by the Institutional Animal Care and Use Committee of Northeastern University.

### Endurance exercise regimen

Female *Polg* ^D257A/D257A^ mice were housed individually in pathogen-free facilities with free access to food and water. Body weight and condition were analyzed weekly throughout the study. At 3 months of age, *Polg* ^D257A/D257A^ were randomly assigned to sedentary (POLG-SED) or endurance exercised (POLG-END) groups. POLG-END mice were exercised three times per week on a mouse treadmill (Exer6M Treadmill, Columbus Instruments) at 15 m/min for 45 min. The exercise protocol was continued from 3 months of age through the duration of the study (up to 9 months of age). Once per month, mice from both the POLG-SED and POLG-END groups were subjected to an endurance stress test, which consisted of running on a treadmill at slowly increasing speed intervals (1 m/min increase every 2 min) until they could no longer run on the treadmill for ten continuous seconds. We were repeatedly unable to retrieve oocytes from POLG mice post 9 months of age due to lack of ovulation following hyper-stimulation, and thus, the study concluded when mice reached 9 months of age, within the normal reproductive time frame for WT C57BL/6 female mice. Not unexpectedly, our pilot studies demonstrated no difference in oocyte quality between exercised C57BL/6 and sedentary C57BL/6 during this time frame (Supplementary Fig. 1, see section on supplementary data given at the end of this article), and so sedentary C57BL/6 were included as a reference for ‘normal’ oocyte quality.

### Oocyte retrieval

Ovulation was induced by intraperitoneal injection of pregnant mare serum gonadotropin (PMSG, 10 IU; Sigma-Aldrich) followed by human chorionic gonadotropin (hCG, 10 IU; Sigma-Aldrich) 46–48 h later. Fifteen to sixteen hours post hCG injection, mice were killed by CO_2_ asphyxiation, and the ovaries and attached oviducts were collected. Oocytes were released from the oviducts by puncturing the oviducts with an insulin syringe, and oocytes were collected via pulled glass pipet. Collected oocytes were denuded of cumulus cells by incubating for 2 min in 80 IU/mL of hyaluronidase (Sigma-Aldrich) at 37°C, followed by three washes with human tubal fluid (HTF; Irvine Scientific, Santa Ana, CA, USA) supplemented with 0.4% BSA (fraction V, fatty acid free; Sigma-Aldrich) at 37°C. Oocytes were counted and classified using a Zeiss Stereo Microscope (Zeiss) as MII (extrusion of the first polar body into the perivitelline space), maturation arrested (germinal vesicle stage, or germinal vesicle breakdown without polar body extrusion) or dead (membrane blebbing, oocyte fragmentation or condensed cytoplasm). Following analysis, MII oocytes were fixed in 2% paraformaldehyde for 30 min at 37°C for endpoint analyses.

### Follicle counting

Following oocyte retrieval, ovaries from 3-, 7- and 9-month-old female mice were collected and fixed, paraffin embedded and serially sectioned (8 μm). The sections were mounted on slides and subsequently stained with hematoxylin and picric methyl blue for the assessment of total, primordial, primary and preantral follicle numbers as described previously (Wang* et al.* 2017).

### Mitochondrial distribution analysis

Mature, MII oocytes were collected, denuded and fixed as described earlier, followed by incubation in permeabilization buffer (1% bovine serum albumin (BSA), 5% normal goat serum (NGS), 0.1% Triton-X, 0.05% Tween-20 in PBS) for 30 min. Oocytes were then stained with 500 nM MitoTracker Red CMXros for 1 h at room temperature. Once stained, oocytes were washed in PBS (Sigma-Aldrich) and then mounted and imaged at 63× magnification on a laser scanning confocal microscope (Zeiss). Mitochondrial distribution was classified as normal upon observing a uniform and distinctly punctate cytoplasmic distribution, whereas oocytes containing mitochondria having diffuse (non-punctate) or condensed mitochondrial distribution were classified as abnormal (Selesniemi* et al.* 2011).

### Immunofluorescence

Fixed MII oocytes were incubated in permeabilization buffer for 30 min, followed by a brief wash in PBS and incubation in blocking buffer (2% BSA, 2% NGS in PBS) for 1 h. A 1:100 dilution of mouse anti α-tubulin antibody (Sigma-Aldrich) was added to the sample, and incubated for 1 h, followed by three washes for 5 min each in PBS. The samples were then incubated with goat anti-mouse conjugated to Alexa-488 (1:500; Life Technologies). The oocytes were then washed three times in PBS for 5 min, with DAPI (1:100) added during the final wash step. Oocytes were then mounted and analyzed by confocal microscopy for spindle morphology and chromosome alignment. Each oocyte was scored based on the structural appearance of the meiotic spindle as well as the arrangement of the chromosomes. Oocytes were marked as normal upon assessment of a barrel-shaped spindle with chromosomes aligned centrally along the metaphase plate. Conversely, oocytes were scored as abnormal following observation of one or more phenotypic defects, including a reduction in, or displacement of, microtubules, failure of microtubules to attach to chromosomes and/or dispersion or misalignment of chromosomes (Selesniemi* et al.* 2011, Ben-Meir* et al.* 2015).

### mtDNA analysis

Following immunofluorescence staining, the fixed MII oocytes were retrieved from slides and incubated in 1 μL lysis buffer (10 mM EDTA, 0.5% SDS, 0.1 mg/mL Proteinase K) under mineral oil for 3 h at 37°C before storage at −80°C. mtDNA was amplified almost in its entirety (16,162 bp) by single molecule PCR (smPCR) with the Q5 Hot Start High-Fidelity Polymerase (New England Biolabs). The DNA samples were serially diluted until an optimal concentration was reached, consisting of 32 reactions, with approximately 1/3 of wells yielding a 16 kb band, with the remaining wells being negative, to ensure single molecule analysis. The subsequent PCR products were individually sequenced in 24 separate Sanger sequencing reactions with overlapping reads for *post hoc* full-length sequence assembly. Six and seven mitochondrial genomes were sequenced from single oocytes of individual representative POLG-END and POLG-SED mice, respectively, using a 3720xl DNA Analyzer (Applied Biosystems). Sequences were analyzed against the C57BL/6J mouse mitochondria reference genome (GenBank ID AY172335.1) using CodonCode Aligner software (CodonCode Corporation). A total of 199,966 bp were sequenced and 213 mutations detected and manually verified. For mtDNA copy number analysis, short, 1 kb fragments were amplified following the single molecule protocol described earlier. Copy number was assessed through Poisson distribution correction (Kraytsberg* et al.* 2009).

### Statistical analysis

Quantitative data from experimental replicates were combined and are presented as the mean ± s.e.m. or mean ± standard deviation (s.d.) of binomial distribution. Analysis of statistical significance was performed using Student’s *t*-test, with *P* values <0.05 considered significant. Statistical comparisons between more than three groups were performed using ANOVA, followed by Tukey’s *t*-test (*P* < 0.05 considered significant). For comparing mutant fractions of mitochondria in oocytes, the ratios of the number of mutations to total bases sequenced were compared, and *z*-score values were used to calculate* P* values.

## Results

**Figure 1:**
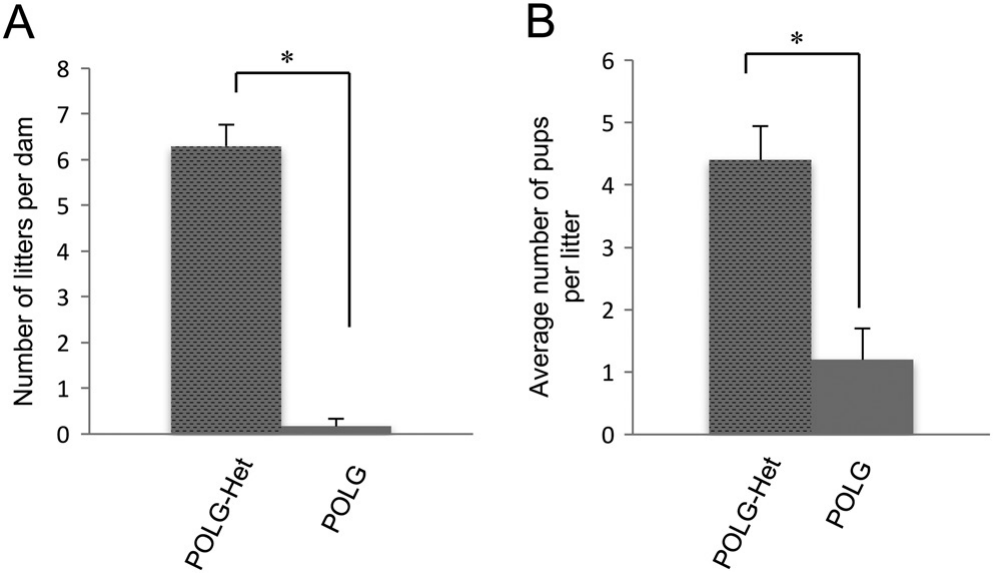
Female POLG mitochondrial DNA mutator mice have reduced fertility as compared to heterozygous littermates. The number of litters per dam (A), and number of pups per litter (B), is shown for mice heterozygous (*Polg* ^D257A/+^; POLG-het) and homozygous (*Polg* ^D257A/D257A^; POLG) for the POLG mutation over a 12-month period, with male-pairing beginning at 6 weeks of age (*n* = 10 female mice per group, *P < 0.001).

It has previously been reported that both male and female *Polg* ^D257A/D257A^ mice have impaired fertility (Trifunovic* et al.* 2004, Hance* et al.* 2005, Kujoth* et al.* 2005, Safdar* et al.* 2011*a*). Our data confirm these reports in females. Notably, female *Polg* ^D257A/D257A^ mice produced no to very few litters between 2 and 9 months of age, with a dramatic reduction in the number of pups per litter as compared to *Polg* ^D257A/+^ animals (Fig. 1A and B). Due to the anticipated and demonstrated dramatic reduction in the number of viable offspring for analysis from *Polg* ^D257A/D257A^ breeding pairs, all *Polg* ^D257A/D257A^ used in this study were obtained from *Polg* ^D257A/+^ × *Polg* ^D257A/+^ crosses.

**Figure 2:**
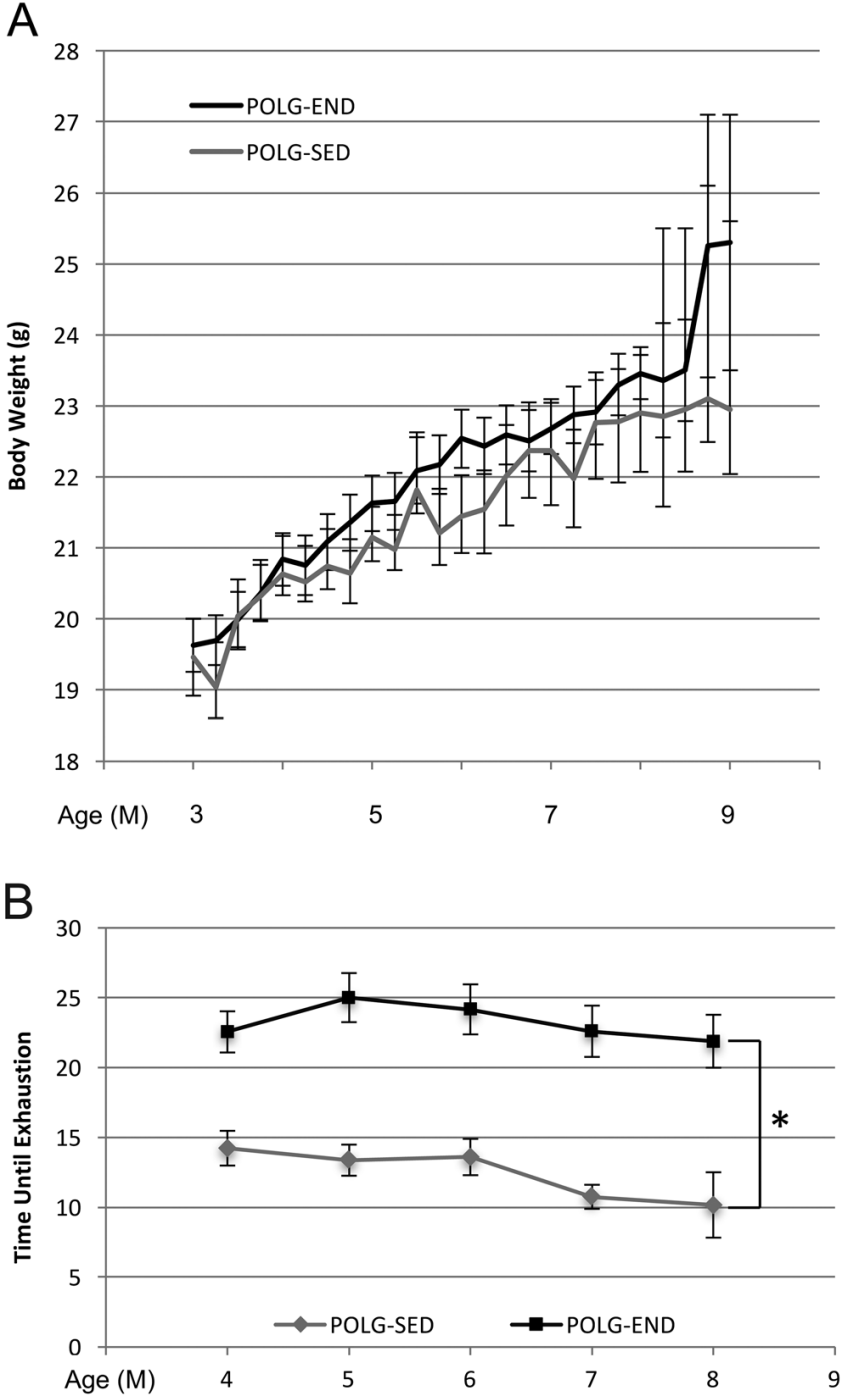
Treadmill endurance exercise three times per week for 45 min at 15 m/min. (A) Body weight (g) for sedentary (POLG-SED) and POLG endurance-exercised (POLG-END) POLG mutator mice 3–9 months (M) of age. (B) Endurance test for POLG-SED and POLG-END mice. Mean ± s.e.m.; *P < 0.01 for POLG-SED vs POLG-END at all time points; *n* = 10 mice per group.

**Figure 3:**
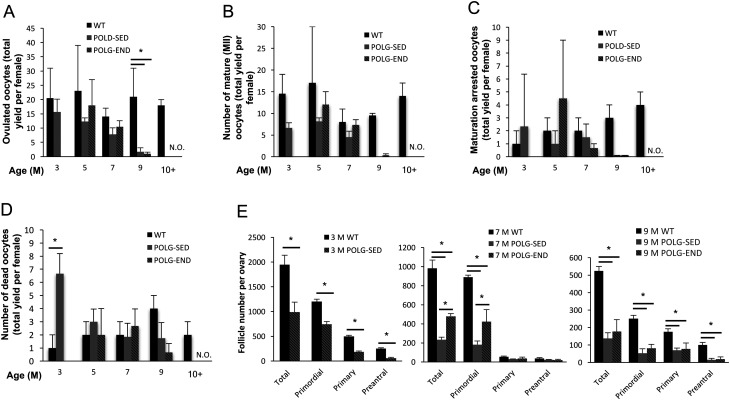
Oocyte numbers in POLG mice following endurance exercise. (A) Yield of oocytes following induced ovulation, collected from 3-, 5-, 7-, 9- and >10-month-old (M) WT, POLG-sedentary (POLG-SED) and POLG endurance-exercised (POLG-END) mice (mean ± s.e.m.; *n* = 4–6 mice per group; N.O., no oocytes ovulated; * indicates significance, *P* = 0.01). (B) Number of mature (MII) oocytes per female (mean ± s.e.m.; *n* = 4–6 mice per group). (C) Number of maturation arrested oocytes per female (mean ± s.e.m.; *n* = 4–6 mice per group). (D) Number of dead oocytes per female. Note: at 3 M endurance exercise treatments have not yet begun, and no oocytes were ovulated from POLG-SED or POLG-END mice post-9 M of age. (E) Enumeration of non-atretic primordial, primary and preantral follicles in the ovaries of 3-, 7- and 9-M WT, POLG-SED, and POLG-END mice. (WT, solid black bars; POLG-SED, solid gray bars; POLG-END, lined gray bars; mean ± s.e.m.; *n* = 4–6 mice per group; *P < 0.05.)

We then sought to determine whether endurance exercise could abrogate the accelerated age-related decline in oocyte quality in *Polg* ^D257A/D257A^ mice. POLG mice were placed into either sedentary (POLG-SED) or endurance-exercised (POLG-END) groups. For endurance exercise training in all experiments, mice were placed on an exercise treadmill and run for 45 min, 3 times per week, for the duration of the experiment. Exercise generated a slight, but not statistically significant, increase in body weight as compared to the POLG-SED group (Fig. 2A). Additionally, when body conditioning was evaluated in an endurance stress test, POLG-END mice were able to consistently spend more time on the treadmill than their POLG-SED counterparts (*P* < 0.01 for 4- to 8-month-old mice). To determine whether exercise improves oocyte yield in POLG mice, WT (control), POLG-SED and POLG-END mice 3, 5, 7 and 9 months of age were superovulated, and the total number of ovulated oocytes from each group were counted and assessed for maturation status. Both total oocyte yield, as well as the number of oocytes that reached MII, were modestly, but not significantly, decreased in POLG mice between 3, 5 and 7 months of age as compared to WT and were similar between the POLG-SED and POLG-END groups at 5, 7 and 9 months of age (Fig. 3A and B). There was a significant decline in the number of ovulated oocytes from POLG-SED and POLG-END as compared to WT at 9 months of age, with no oocytes ovulated from either POLG group thereafter (Fig. 3A, B, C and D). Additionally, there were no quantitative differences in the number of maturation arrested or dead oocytes between the groups, up to 7 months of age, with the dramatic decline in the number of ovulated oocytes at 9 months of age making the POLG groups no longer comparable to WT. However, there were no detectable differences between the POLG-SED and POLG-END groups (Fig. 3C and D). In order to determine if endurance exercise impacts the number of follicles in the ovary, ovaries were collected from mice at 3, 7 and 9 months of age, and the number of total, primordial, primary and preantral follicles was determined. The reserve of oocyte-containing primordial follicles in the ovaries of POLG mice was significantly diminished as compared to WT mice at all ages examined (Fig. 3E). Exercise did prevent the decline in the primordial follicle pool in POLG mice from the 7-month-old age group; however, this trend was not sustained in the 9-month-old POLG-END mice. Taken together, these data suggest that while exercise may prevent early loss of follicles in *Polg* ^D257A/D257A^ mice, it does not sustain follicle numbers past 9 months of age, nor does it impact the number or maturation status of ovulated oocytes.

**Figure 4:**
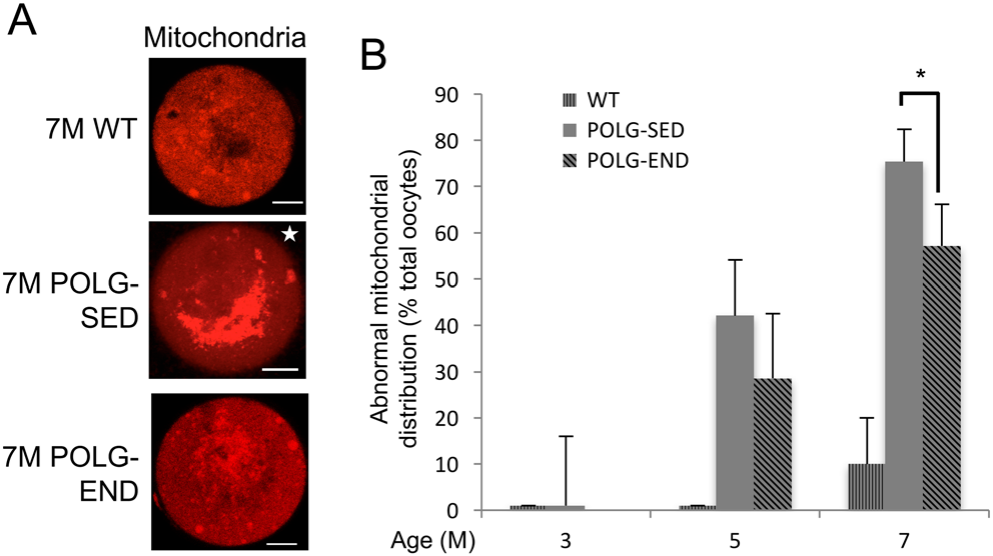
Exercise maintains normal mitochondrial distribution in oocytes from POLG mice at 7 months of age. (A) Representative images of MII eggs labeled with MitoTracker (shown in red) from 7-month-old (7 M) WT, POLG-sedentary (POLG-SED), and POLG endurance exercised (POLG-END) mice (mitochondrial staining with MitoTracker shown in red). Star indicates abnormal mitochondrial patterning; scale bar = 15 μm. (B) Occurrence of abnormal mitochondrial distribution in MII oocytes of WT, POLG-SED and POLG-END mice (mean shown, error calculated as s.d. of a binomial distribution; *n* = 20–49 oocytes from 5–6 mice per group; *P < 0.05).

We next sought to determine if exercise impacts the quality of MII oocytes in POLG mice. Previous work has demonstrated that mitochondrial aggregation is linked to the age-associated decline in oocyte quality (Selesniemi* et al.* 2011). Evaluation of mitochondrial distribution in MII oocytes using MitoTracker labeling followed by confocal microscopy and assessment of mitochondrial distribution demonstrated that by 5 months of age, POLG-SED mice exhibited significant aberrant mitochondrial distribution as indicated by mitochondrial aggregation and clustering, as compared to WT controls, which largely demonstrated even, punctate patterning. In this age group, exercise had little impact on the mitochondrial distribution in oocytes from POLG mice. However, by 7 months of age, the aberrant mitochondrial phenotype was more pronounced in the POLG-SED group; an effect which was partially abrogated by exercise (Fig. 4A and B).

**Figure 5:**
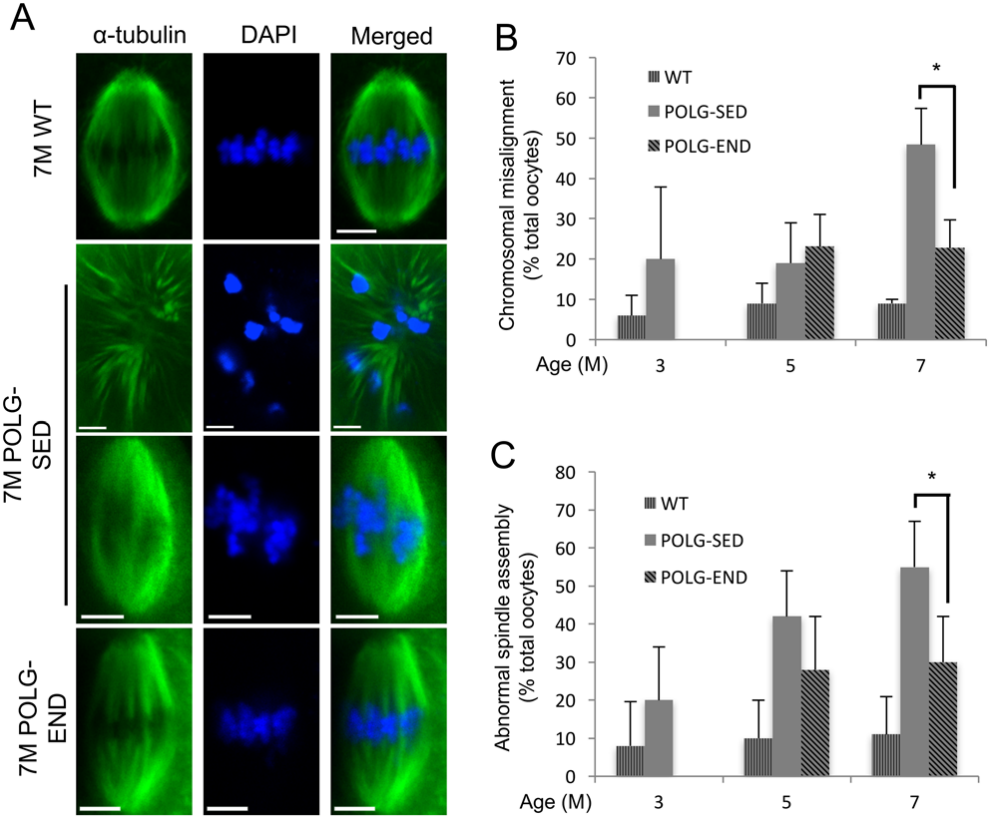
Exercise improves meiotic spindles and chromosome alignment in POLG mice at 7 months of age. (A) Representative laser scanning confocal micrographs of meiotic spindles in MII oocytes from 7-month-old (7 M) WT, POLG-sedentary (POLG-SED) and POLG endurance-exercised (POLG-END) mice; α-tubulin labeling of spindle (green) and DNA labeled with DAPI (blue). Scale bars = 5 μm. (B and C) Occurrence of spindle abnormalities and chromosome misalignment in oocytes from 3 M WT and POLG-SED, and 5 and 7 M WT, POLG-SED and POLG-END mice (mean shown, error calculated as s.d. of a binomial distribution; *n* = 20–39 oocytes from 5–6 mice per group; *P < 0.05).

A parallel pattern was observed for meiotic spindle integrity and chromosomal arrangement (Fig. 5A, B and C). Following confocal microscopy and analysis of spindle formation and chromosomal alignment, it was demonstrated that in mature, MII oocytes collected from WT (control), POLG-SED and POLG-END females, the number of spindle abnormalities was unchanged in the POLG mice at 3 months of age as compared to WT, and by 5 months of age, little difference was observed between the POLG-SED and POLG-END groups. However, by 7 months of age, MII oocytes collected from POLG-SED mice exhibited significant spindle malformation as compared to oocytes from WT mice, whereas the number of abnormalities observed in POLG-END mice were unchanged from the levels quantified at 5 months of age (Fig. 5A, B and C). Together, these data indicate that exercise may, at least in part, abrogate the oocyte abnormalities associated with poor oocyte quality in POLG mice.

**Figure 6:**
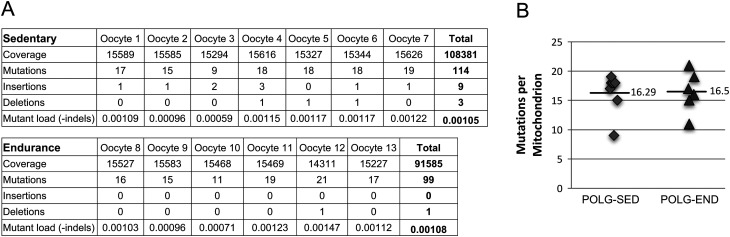
Exercise does not impact mitochondrial mutations in oocytes from POLG mice. (A) mtDNA sequencing coverage, numbers of mutations, insertions and deletions, and total mutational load in single oocytes from individual sedentary (*n* = 7 oocytes) and endurance-exercised (*n* = 6 oocytes) 7-month-old POLG mice. (B) Average mutational number in POLG-sedentary (POLG-SED) and POLG endurance-exercised (POLG-END) mice. Mean ± s.e.m.; no significant change in mutational number (*P* = 0.91) or mutational load (*P* = 0.80).

To determine if the improvement in oocyte quality observed in oocytes from 7-month-old POLG females following exercise could be attributed to, in part, to a reduction in mitochondrial mutational load, we compared the number of mitochondrial mutations in individual fixed MII oocytes demonstrated to have normal mitochondrial distribution (observed by MitoTracker) in POLG-SED (*n* = 7) and POLG-END (*n* = 6) mice. Using single molecule PCR (smPCR) and Sanger sequencing, we determined the mtDNA mutant fraction for each group; POLG-SED had an average mutant fraction of 1.08 × 10^−3^ (the equivalent of ~17.6 mutations per genome) while POLG-END had a mutant fraction of 1.05 × 10^−3^ (~17.1 mutations per genome). We found no discernible difference in mitochondrial mutational load between the POLG-SED and POLG-END groups (*z*-score = −0.199, *P* value = 0.841) (Fig. 6A and B). Additionally, to determine whether the observed deficits in female fertility are linked to a defect in mitochondrial biogenesis, we evaluated mtDNA copy number in WT, POLG-SED and POLG-END mice at 9 months of age and detected no appreciable differences in mtDNA copy number across the groups (data not shown). Collectively, these data indicate that while exercise does not impact oocyte numbers or maturation status in POLG mice, exercise does confer a modest benefit on oocyte quality that is independent from mitochondrial mutational load.

## Discussion

The rapid accumulation of mutations generated by the defect in the proofreading-exonuclease activity of POLG in the mtDNA mutator mouse leads to a number of phenotypic defects across somatic tissues that are frequently associated with aging. By 7–9 months of age, POLG mice exhibit sarcopenia, depletion of adipose tissue, cardiomyopathy, fur loss and graying and other hallmark features of the aging process (Trifunovic* et al.* 2004, Kujoth* et al.* 2005). Consistent with these findings, data herein establish that ovarian function at the level of the oocyte are prematurely diminished in the POLG mouse, as demonstrated by an accelerated reduction in the number of primordial follicles, aberrant mitochondrial distribution in oocytes and an increase in spindle malformation and chromosomal misalignment, similar to that observed in aged mice (Selesniemi* et al.* 2011). Additionally, we report that exercise performed in moderation (45 min, 3 times per week) is sufficient to modestly improve phenotypic markers of oocyte quality in POLG mice; however, it does not result in an extended time frame for fertility. By 9 months of age, we were unable to consistently obtain oocytes for analysis from either sedentary or exercised POLG mice. Importantly, although modest improvements in oocyte quality occurred following exercise, the mitochondrial mutational rate remained unchanged in the oocytes of exercised POLG mice, as compared to their sedentary counterparts similar to what has been reported in muscle (Safdar* et al.* 2011*a*,*b*, 2016*b*).

Illustrating the difference between oocytes and somatic cells, it has recently been demonstrated that a similar endurance exercise protocol leads to reduction in the mtDNA non-mutational damage burden in skeletal muscle, largely through a p53-dependent mechanism in which p53 is localized to mitochondria and acts as an accessory fidelity-enhancer to the DNA-repair mechanism of POLG, forming a complex together with Tfam at the level of mtDNA (Safdar* et al.* 2016*b*). Additionally, in somatic tissue exercise induced the translocation of nuclear p53 to mitochondria, which in turn lead to upregulation of peroxisome proliferator-activated receptor gamma, coactivator 1 alpha (PGC1-α), an important modulator of metabolism and mitochondrial biogenesis (LeBleu* et al.* 2014, Safdar* et al.* 2016*b*). In oocytes, however, the positive impact of exercise on oocyte quality is likely governed by alternative mechanisms. For example, while mice deficient in PGC1-α exhibit increased offspring mortality, females that do survive to 12 months of age demonstrate improved markers of oocyte quality, including dramatically reduced spindle abnormalities and chromosomal misalignment, as well as a decrease in abnormal mitochondrial aggregation as compared to WT. These data indicate that lack of PGC1-α has a positive, rather than detrimental, impact on oocyte quality with age (Selesniemi* et al.* 2011). Furthermore, while a fidelity-enhancing mechanism for p53 following exercise has been proposed for somatic tissues by ourselves and others (Safdar* et al.* 2016*a*,*b*), it is unlikely that this is the case in oocytes, as the role of p53 and its related family members, p63 and p73, are unique in oocytes as compared to somatic cells types (Hu* et al.* 2007, Levine* et al.* 2011). Given distinct role of the p53 family members in oocyte function, it is likely that, similar to PGC1-α, the molecular mechanisms that mediate the impact of exercise on oocyte quality are divergent from those operating in the soma. It should be noted that even the basic processes of biogenesis and mitophagy are dissimilar in oocytes, with biogenesis halted prior to development to MII and mitophagy thought to be absent (Boudoures* et al.* 2017). Importantly, mitochondrial biogenesis in POLG mice is associated with an increase in mtDNA mutational load (Dillon* et al.* 2012), as mitochondrial genomic replication with a faulty polymerase results the steady accumulation of mutations. In this study, exercised POLG mice did not accrue an additional mutational burden as compared to their sedentary counterparts. Together, these data indicate that exercise does not result in appreciable mitochondrial biogenesis, beyond what is observed in sedentary counterparts, in the oocytes of POLG mice.

Nonetheless, while mitochondrial mutational load was unaffected, moderate exercise did have a modest positive impact on oocyte quality during the latter stages of the fertile period (prior to 9 months of age) in the POLG mouse. The exercise regime included treadmill exercise at regular intervals throughout the week, in addition to an endurance stress test performed once per month. The endurance exercise group consistently exceeded the sedentary group, with a significant increase in the time to exhaustion. Thus, the total exercise regime includes regular moderate exercise with intervals of more strenuous physical activity. Although the total number of MII oocytes collected was not significantly greater than sedentary counterparts, the metrics for quality in oocytes from exercised POLG mice were improved. However, the impact of exercise on oocyte quality was observed only at the 7-month time point. At 7 months, oocytes from sedentary POLG mice showed elevated defects in spindle assembly, chromosome alignment and mitochondrial distribution, whereas oocytes from exercised POLG mice maintained the profile from earlier time points (3 and 5 months). Following 7 months, oocytes were infrequently obtained from either sedentary or exercised POLG mice. Together, these data collectively indicate that exercise has a modest effect on oocyte quality, with a lack of impact on total oocyte numbers and does not extend the fertile lifespan in female POLG mice. This is, again, in stark contrast to what has been reported in somatic tissues in the POLG mouse, in which the benefit of exercise is pronounced.

These findings are in agreement with a recently published study in which voluntary exercise induced moderate improvements on the structure of mitochondria in oocytes of mice fed a high-fat diet (HFD), reducing the number of rose-petal and elliptical-shaped mitochondria (Boudoures* et al.* 2016). Additionally, the oocytes of exercised mice fed a HFD had reduced lipid accumulation. However, unlike our study, which utilized a forced endurance exercise protocol, oocytes from the HFD mice on a voluntary exercise regime did not exhibit improvements in MII spindle structure. Although both studies demonstrate modest improvements in oocyte quality following moderate exercise protocols, exercise alone was not sufficient to rescue oocyte quality to levels comparable to control or WT mice. In women, the impact of exercise on the timing of menopause remains a topic of debate. A number of studies have examined the correlation between exercise status and the age at menopause in women, with contradictory results (Cumming* et al.* 1994, Cassou* et al.* 1997, Amigoni* et al.* 2000, Cooper* et al.* 2001). While moderate exercise does not appear to have influence on menopausal age (Bromberger* et al.* 1997, Amigoni* et al.* 2000, Palmer* et al.* 2003), excessive exercise (>8 hours per week) is potentially linked to a decrease in the age of onset of menopause (Cumming* et al.* 1994, Nagata* et al.* 2012).

The benefit of exercise on both extension of lifespan as well as a reduction in instances of chronic diseases has been well documented in both humans and animal models (Chakravarty* et al.* 2008, Stessman* et al.* 2009). In humans, even moderate regular exercise significantly increases lifespan (Moore* et al.* 2012, Arem* et al.* 2015). In rodents, it has been demonstrated that exercise results in a decrease in the instance of chronic illness (Garton* et al.* 2016). Most notably, moderate endurance exercise has the remarkable effect to systemically ameliorate the many age-associated phenotypic characteristics of the POLG mutator mouse and modulate the metabolic profile of the brain (Safdar* et al.* 2011*a*,*b*, 2016*b*, Clark-Matott* et al.* 2015). However, based on the results reported herein, the benefit of exercise on female fertility at the level of the oocyte in the POLG mouse is relatively limited as compared to somatic tissues, which is likely indicative of the highly specialized molecular mechanics of the oocyte, which, unlike somatic tissues, give rise to subsequent generations through eventual propagation of the germline. Although it is possible that this is a limitation of the model itself, in that the high mutational burden simply might not be countered by exercise, it is intriguing that the intervention that so widely impacts the somatic tissues does not confer a similar benefit to oocytes in the POLG mouse and demonstrates yet another paradigm in which the oocyte differs mechanistically in dramatic fashion as compared to somatic cells and tissues. This finding highlights functional features, appearing to be exclusive to oocytes, which mediate mitochondrial biology. Accordingly, it remains likely that further investigations into the molecular mechanisms that uniquely govern mitochondrial function in the oocyte utilizing the POLG mouse model will uncover additional alternative features of mitochondria specific to the germline.

## Supplementary Material

Supporting Figure 1

## Declaration of interest

C F, J J and H L declare no competing financial interests. D C W discloses interest in intellectual property described in U.S. Patent 8,642,329, U.S. Patent 8,647,869 and U.S. Patent 9,150,830 and is a recipient of a corporate sponsored research award from OvaScience, Inc. K K, S A and D C W declare interest in intellectual property described in United States Patent Application No. 62/273,702, under examination.

## Funding

This material is based upon work supported by the National Science Foundation under grant number 1750996 to D C W and by the Ellison Medical Foundation Senior Scholar Award to K K.
