# Supplementary figures and images for: Impact of exercise on oocyte quality in the POLG mitochondrial DNA mutator mouse
Source: Reproduction. 2018 Jun 5;156(2):185–94. doi: 10.1530/REP-18-0061 (PMC6074767; doi:10.1530/REP-18-0061)

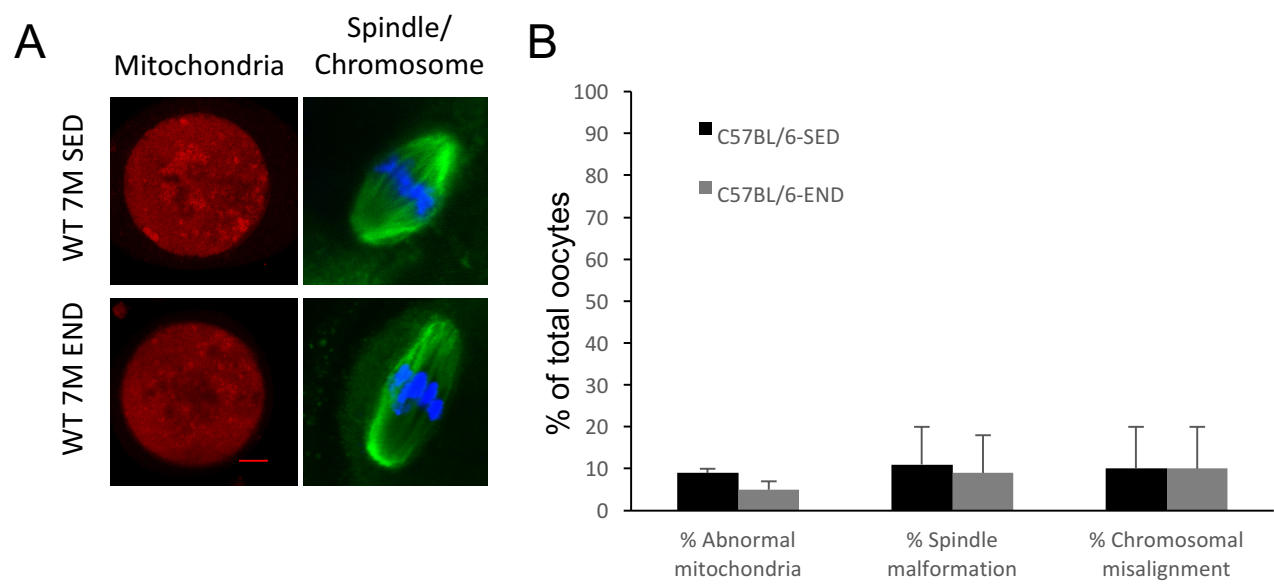

**Supplemental Figure 1**

Supplement: Supporting Figure 1 [file rep-156-185-s001.pdf]
